# Rare and unexpected ventilation difficulties due to tracheal diverticulum: A case report

**DOI:** 10.1097/MD.0000000000034536

**Published:** 2023-08-11

**Authors:** Weiwei Zhou, Yantao Lang, Zhongling Xu, Dekun Yin

**Affiliations:** a Department of Anesthesiology, Affiliated Hospital of Nantong University, Nantong, China; b Department of Anesthesiology, Funing People’s Hospital of Jiangsu, Yancheng, Jiangsu Province, China.

**Keywords:** fiberoptic bronchoscopy, tracheal diverticulum, tracheotomy

## Abstract

Tracheal diverticulum is a rare airway-related particular occurrence, and the forcible tube insertion may cause tracheal ruptures during tracheotomy. Therefore, fiberoptic bronchoscopy (FOB) should be used routinely on all patients undergoing tracheal intubation or tracheotomy.

**Patient concerns:**

A 60-year-old male with laryngeal neoplasms was scheduled for partial laryngectomy using a suspension laryngoscope in July 2020. All operations were performed under general anesthesia through orotracheal intubation. Orotracheal intubation was a noninvasive procedure that could effectively control breathing. At the end of the surgery, the percutaneous tracheostomy was performed to maintain airway patency, facilitate spontaneous respiration, and remove the secretions.

**Diagnoses:**

At this moment, the tracheal diverticulum, located at the right posterolateral region of the trachea, became an unexpected airway-related particular occurrence, which led to tracheal tube placement difficulty, mechanical ventilation difficulty, and high airway pressure.

**Interventions:**

Subsequently, the tracheal tube was repositioned, with placement again confirmed by the FOB.

**Lessons subsections:**

Tracheal diverticulum is an infrequent cause of tube inserting difficulty for the tracheotomy, and FOB is the first option for patients with catheter placement difficulty and mechanical ventilation difficulty.

## 1. Introduction

Paratracheal air cyst is a rare entity that usually arises close to the trachea, including tracheocele, tracheal diverticula, and bronchogenic cysts. It is estimated that there are approximately 3.7% to 8.1% of patients with paratracheal air cysts,^[1,2]^ and only 14% are detected by chest radiographs.^[3]^ Tracheal diverticulum (DV), arising from the posterior tracheal wall where the cartilage rings are incomplete, is a benign cyst-like lesion filled with a large amount of secretion (sputum and saliva), which might cause the symptoms of cough, dyspnea, stridor, and chronic chest infection.^[4,5]^ Although it is rarely encountered in a clinical setting, Marina Pace et al^[6]^ reported that a total of 124 DV was detected by computed tomography (CT) in 1679 patients, resulting in a prevalence of 7.4%, and the incidence is approximately 2 times higher in men compared with women. Tracheal DVs are rarely located at the left side (2.9%), and usually locate at the right posterolateral region of the trachea (97.1%),^[7]^ especially a few centimeters above the tracheal bifurcation. This region has little effect on orotracheal intubation but is critical for the tracheotomy because of a close distance from the incision, which increases the potential risk of tracheotomy, such as tracheal tube placement difficulty and subsequent ventilation difficulty, hypoxemia. Although fiberoptic bronchoscopy (FOB) remains the golden standard for managing percutaneous tracheostomy, there are many difficulties and high clinical risks in the tracheal tube placement under bronchoscopic guidance because of potential bleeding states, unclear anatomical hierarchy, misdirection into interstitial space. Meanwhile, for the acquired tracheal DV combined with a weakened musculature, tracheal diverticulum breakage, induced by a forcible tube insertion or bronchoscopic guidance during the operation for tracheotomy, might put patients in serious condition. Therefore, careful evaluation of preoperative CT and percutaneous tracheostomy is necessary to avoid injury of the tracheal diverticulum.

In this case report, we present rare and unexpected tracheal tube placement and ventilation difficulties due to tracheal diverticulum for the patient undergoing percutaneous tracheostomy.

## 2. Case description

**Figure 1.**
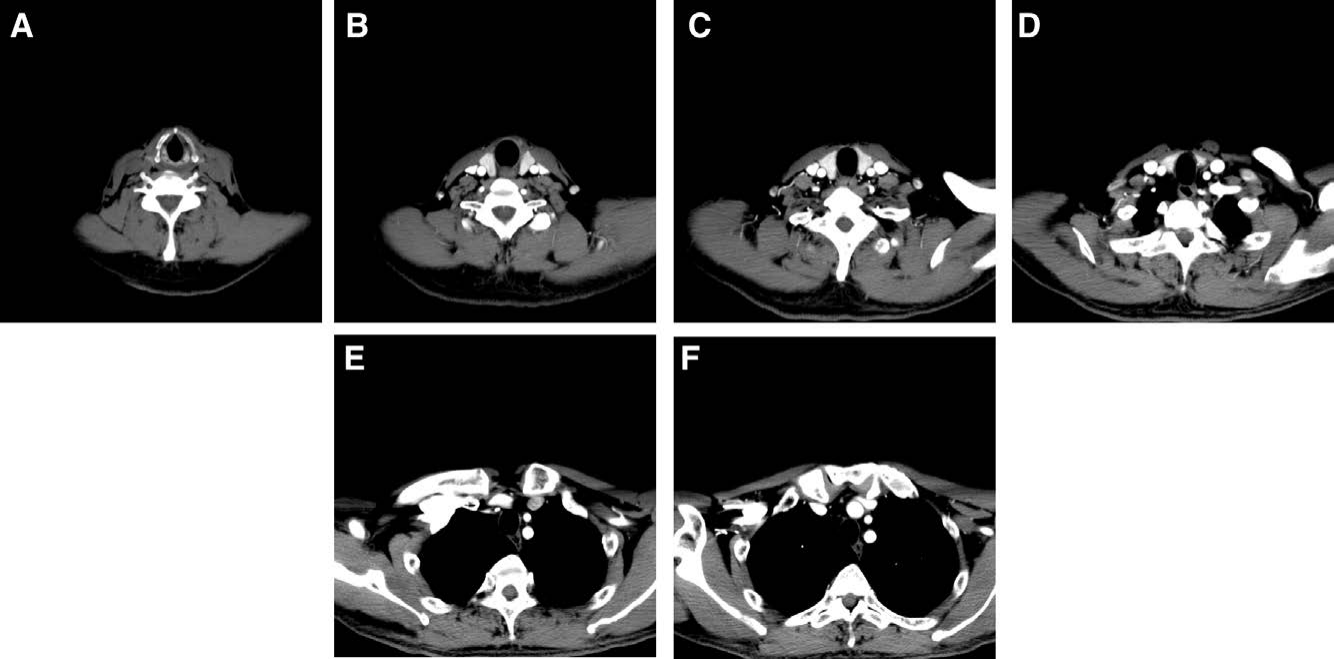
Preoperative neck and chest CT did not reveal a tracheal diverticulum (Fig. 1 A–F). CT = computed tomography.

A 60-year-old male, 171 cm tall and weighing 55 kg, was admitted to the hospital with the chief complaint of pharyngeal discomfort and hoarseness of voice for half a year. The patient had only occasional mild coughing and sputum, without dyspnea, dysphagia, or other pulmonary-related diseases (chronic bronchitis, emphysema, pulmonary heart disease, etc.). The patient had a history of heavy alcohol consumption for over 20 years and smoking 20 cigarettes daily for 40 years. In addition, he had well-controlled diabetes and no hypertension. No noticeable abnormalities were detected on electrocardiograph or cardiopulmonary function examination; and blood biochemistry, hematology, urine of this patient were within the acceptable range, including normal renal and liver function. Contrast-enhanced CT and laryngoscopy only revealed a 22.5*24.5 mm soft-tissue density mass occupying supraglottic levels of the anterior laryngeal wall; tracheal diverticulum was not detected on enhanced CT (Fig. 1A–F). Pathology of the laryngeal biopsies revealed atypical squamous epithelial proliferation and local carcinosis. This patient was scheduled to receive the partial laryngectomy under suspension laryngoscope for a laryngeal malignant tumor in July 2020.

Before surgery, the anesthesia scenarios and surgical procedures were explained to the patient clearly. Operations would be performed under general anesthesia with orotracheal intubation, including the resection of the laryngeal mass, rapid frozen pathological examination, and subsequent radical resection of laryngeal carcinoma. At the end of the surgery, tracheotomy and intubation must be performed in the neck to avoid acute respiratory obstruction induced by the incision hemorrhage or laryngeal edema. The other anesthesia scenarios (potential impending dangers and response measures, etc) were also explained to the patient and his family, then they agreed and signed informed consent forms for anesthesia.

In the operating room, the patient’s vital signs were monitored, and the data were within the acceptable range, including oxygen saturation which was 98%. After 3 minutes of general anesthesia induction with propofol, fentanyl and cisatracurium, transoral intratracheal intubation was performed uneventfully with an insertion depth of approximately 22cm, and the patient was ventilated in a volume-controlled mode with a frequency of 12 to 14 breaths/minutes and a tidal volume of 8 to 10 mL/kg. All ventilation-related parameters in the anesthesia machine were within acceptable ranges. During the operation, high airway pressure (28–35 mm Hg) occurred occasionally and was effectively relieved by sputum suction, adjusting the tube depth, and lowering tidal volume. The anesthetic procedure was uneventful, without any particular conditions for the patient.

**Figure 2.**
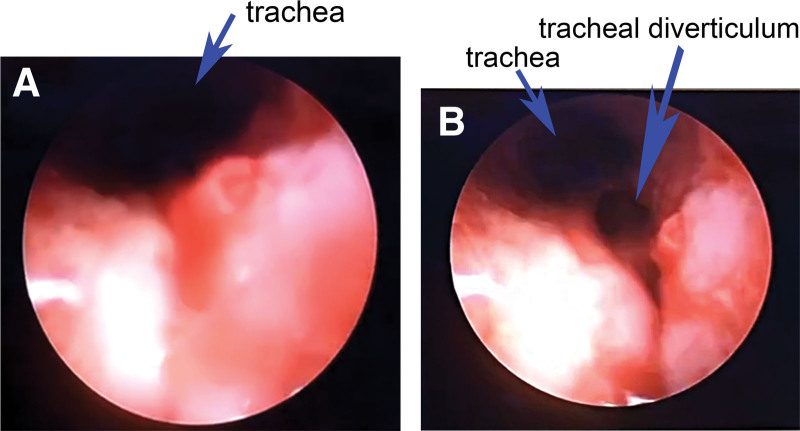
Fiberoptic bronchoscope shows a tracheal diverticulum at the right posterolateral region of the trachea and approximately 2 centimeters above the tracheal bifurcation (Fig. 2 A and B).

After the operation, the percutaneous tracheostomy was performed to avoid postoperative ventilation difficulty or dyspnea caused by the incision hemorrhage or laryngeal edema when awakening during the recovery period. When the ventilation catheter was inserted through the neck incision, several special conditions were presented, including tracheal tube placement difficulty, high airway pressure, and ventilation difficulty. After passing through the endotracheal tube, the fiberoptic bronchoscope was advanced into the trachea, and revealed that the tube was inserted into a tracheal diverticulum at the right posterolateral region of the trachea and approximately 2 centimeters above the tracheal bifurcation (Fig. 2A and B). Subsequently, the tracheal tube tip was repositioned and placed distal to the opening of the tracheal diverticulum by the FOB. Meanwhile, we carried out a series of preparations on bronchial intubation, and contacted the thoracic surgery team that was on call. All doctors reached a consensus: the patient would expose to great potential risks of airway complications and even mortality when he accepted the partial laryngectomy, percutaneous tracheostomy, and tracheal DV-related operations simultaneously. Therefore, we considered all special conditions and prepared the corresponding reliability measures to cope with tracheal diverticulum ruptures, such as patch tracheoplasty. The patient was eventually extubated without incident a few days later, and transferred to the thoracic surgery ward the next day. When being followed up after several days of recovery, informed consent was obtained from the patient to publish this case report details.

## 3. Discussion

The tracheal diverticulum, divided into congenital and acquired, was a benign condition characterized by single or multiple cavities lined by ciliated columnar epithelium.^[8]^ The congenital type is considered to be a malformed supernumerary lung or an aborted, abnormally high division of the primary lung bud, and generally consists of respiratory cartilage, smooth muscle and epithelium.^[5]^ It has a smaller diameter and a narrow connection to the trachea, which commonly arise on the right side of the trachea and approximately 4 to 5 cm below the true vocal folds or a few centimeters above the tracheal bifurcation.^[5]^ On the other hand, the acquired type is usually associated with chronic bronchopulmonary disease, which is devoid of mucous glands, smooth muscles, and cartilage.^[5]^ It can be further classified into pulsion and traction types, of which, the traction type is caused by inflammation in the thoracic cavity.^[9]^ The pulsion type is caused by the intraluminal pressure increasing induced by chronic cough, and obstructive pulmonary disease combined with the vulnerability of the bronchial wall.^[9]^ Acquired tracheal DV may arise at any level, which is typically wide-mouthed and larger than congenital DV.^[8]^ In our case, the patient has long-term smoking and a history of chronic cough and expectoration. The fiberoptic bronchoscope revealed that a tracheal diverticulum presented a wide mouth and sizeable cystic structure at the right posterolateral region of the trachea and approximately 2 centimeters above the tracheal bifurcation, which opening and closing with the breathing amplitude instead of persistent expanding. Based on those mentioned above, our case is considered a pulsion type tracheal diverticulum. The most likely explanation for this is that increased tracheal intraluminal pressure, caused by chronic cough combined with a weakened musculature of the trachea wall due to repeated respiratory infections, can lead to the acquired form of tracheal DV.^[8,10]^ For the acquired tracheal diverticulum with dilated and weakened tracheal wall, the endotracheal tube or fiberoptic bronchoscope insertion by force could lead to tracheal ruptures and other severe iatrogenic complications.^[11]^ Moreover, the mechanical ventilation-related parameters were also essential for the acquired tracheal DV, and ventilation with high volume or pressure may cause a greater dilation in the airways and DVs, which is possibly one of the important reasons to result in a massive DV opening and a giant DV body, such as our patient. Therefore, it is necessary to evaluate the preoperative CT and percutaneous tracheostomy carefully, preparing and formulating a series of trachea and lung protective measures for unexpected special cases that may occur during periprocedural period.

Preoperative diagnosis of tracheal diverticulum plays an irreplaceable role in preventing anesthesia risks. CT is the most common method for diagnosing tracheal DV, and evaluating its localization, size, contour, and wall thickness,^[8]^ which also can be useful for distinguishing between congenital and acquired lesions according to the presence or absence of cartilage and the size of DV’s neck.^[10]^ The typical CT findings of tracheal DV include a thin-walled air sac at the paratracheal area with or without communication to the tracheal lumen.^[8,12]^ Because of a vast capsular opening and body under the fiberoptic bronchoscope, tracheal DV should theoretically be observed by the preoperative CT in our patient. The reality, though, is very different, which only indicated laryngeal mass, without tracheal DV (Fig. 1A–F). Dynamic CT studies show expansion of a diverticulum during forced expiration and shrinkage during inspiration.^[5]^ Therefore, the most likely reason for this was that the tracheal DV collapse may occur rapidly under the negative airway pressure when the patient was instructed to take a deep breath and hold his breath to facilitate observing the lung tissue using CT. Of course, we also do not preclude some other possibilities, such as direct iatrogenic injury.

Theoretically, preoperative flexible bronchoscopy was the definitive method for diagnosing tracheal diverticulum. Considering the lack of specific subjective symptoms and clinical signs in this case, flexible bronchoscopy was not a routine part of the preoperative examinations. It is often used as a further examination measure when imaging suggests may be present with lesions, and the non-preferred 1. Although this patient had no apparent respiratory-related disease, the cough and expectoration were persistent and misunderstood as being caused by heavy smoking. Clinically, all patients received preoperative radiological evaluation consisting of a chest X-ray or/and CT scan. It had been proposed that the tracheal diverticulum could be visualized using CT scans except for X radiographs, which could not be used to identify cystic distention without calcium deposition.^[9,10,13]^ However, a recent survey showed that 9 of 26 patients (34.6%) were observed a tracheal diverticulum through CT scans,^[1]^ and the rate of accurate initial diagnosis could undoubtedly be significantly improved in CT and 3-dimensional reconstruction.^[9,13,14]^

Ventilation difficulty, one of the most life-threatening exceptional circumstances, could occur in mechanical ventilation after general anesthesia intubation due to the following reasons: airway hyperresponsiveness to various stimuli (such as atracurium-induced histamine), the tracheal tube straying into the trachea or esophagus, blockage of the tube by secretions, bending and folding of tube.^[15–17]^ The effective treatment methods to alleviate high airway pressure mainly include sputum suction, tracheal dilation drugs, tube depth adjustment, and the correct tube position through lung auscultation. Additionally, rare reasons were also thought to be associated with ventilation difficulty such as collapse of upper airways,^[18]^ primary airway narrowing,^[19]^ tracheoesophageal fistula,^[20]^ tracheal diverticulum. In this patient, high airway pressure (30–35 mm Hg) occasionally occurred during the operation and was effectively relieved by sputum suction, adjusting the tube depth, and lowering tidal volume. The anesthetic procedure was uneventful without 1 particular condition for our patient. The possible explanation is that the endotracheal tube tip was far from the tracheal DV, and the signs and symptoms were not evident. However, there were a series of special conditions for tracheal catheter placement when percutaneous tracheostomy because of a tracheal diverticulum at the right posterolateral region of the trachea and approximately 2 centimeters above the tracheal bifurcation (Fig. 2A and B). According to the patient’s physical state, age, and symptoms, the strategy of therapy which may be selected consists of surgical resection, endoscopic cauterization with laser or electrocoagulation, and conservative management (antibiotics, mucolytic agents, and physiotherapy).^[10]^ Considering the partial laryngectomy and percutaneous tracheostomy simultaneously, there were large trachea and ventilation-related risks for further surgical resection intervention on the tracheal diverticulum. The tracheal tube tip was placed distal to the opening of tracheal DV by the FOB, and a series of preparations on bronchial intubation and other emergency measures were ready to ensure the patient’s safety in any place and at any time. The patient’s vital signs were stable, and the patient was eventually extubated without incident a few days later.

## 4. Conclusion

In summary, we should pay attention to the examination of chest CT scans and 3D reconstruction for patients with long-term cough and expectoration to determine lung-related diseases or other special conditions, such as tracheal diverticulum. Definitive preoperative diagnosis and adequate anesthesia preparation greatly reduce the risk of adverse airway events during the perioperative period.

What is more, bronchoscopy should be the first choice for high airway pressure and ventilation difficulty after anesthesia, and we recommend using FOB during the insertion of percutaneous tracheostomy.

## Acknowledgments

We appreciate the hospital staff for their support and assistance and thank them for the understanding and cooperation of the participants.

## Author contributions

**Project administration:** Weiwei Zhou, Dekun Yin.

**Software:** Zhongling Xu.

**Supervision:** Dekun Yin.

**Writing – original draft:** Weiwei Zhou, Yantao Lang, Zhongling Xu.

**Writing – review & editing:** Dekun Yin.
